# Celecoxib activates autophagy by inhibiting the mTOR signaling pathway and prevents apoptosis in nucleus pulposus cells

**DOI:** 10.1186/s40360-022-00633-y

**Published:** 2022-12-01

**Authors:** Weisin Chen, Miersalijiang Yasen, Hanquan Wang, Chenyang Zhuang, Zixiang Wang, Shunyi Lu, Libo Jiang, Hong Lin

**Affiliations:** 1 Department of Orthopaedics, Zhongshan Hospital of Fudan University, 180 Fenglin Road/1609 Xietu Road, 200032 Shanghai, China; 2 Department of Orthopedic Surgery, Zhongshan Hospital Xiamen Branch, Fudan University, 668 Jinhu Rd, District of Huli, Fujian 361015 Xiamen, China; 3 Department of Orthopaedics, Shanghai Geriatric Medical Centre, Fudan University, Shanghai, China

**Keywords:** Celecoxib, Intervertebral disc degeneration, Autophagy, Apoptosis, mTOR

## Abstract

**Background:**

Intervertebral disc degeneration results from a variety of etiologies, including inflammation and aging. Degenerated intervertebral discs feature down-regulated extracellular matrix synthesis, resulting in losing their ability to retain water and absorb compression. Celecoxib is a well-known selective cyclooxygenase-2 inhibitor for treating arthritis and relieving pain. Nevertheless, the mechanism of Celecoxib for treating inflammation-related intervertebral disc degeneration has not yet been clarified.

**Method:**

Protein synthesis was analyzed by western blot. Fluorescent probes DCFH-DA and MitoSox Red detected reactive oxygen species and were measured by flow cytometry. The activity of the kinase pathway was evaluated by protein phosphorylation. Autophagy was monitored by mRFP-GFP-LC3 transfection and LC3 analysis. Mitochondrial apoptotic proteins were analyzed by western blot and cell membrane integrity was measured by flow cytometry. The autophagic gene was silenced by siRNA.

**Results:**

In this study, interleukin-1β stimulation reduced the synthesis of aggrecan, type I and II collagen and caused excessive production of reactive oxygen species. We looked for a therapeutic window of Celecoxib for nucleus pulposus cells to regain extracellular matrix synthesis and reduce oxidative stress. To look into nucleus pulposus cells in response to stimuli, enhancement of autophagy was achieved by Celecoxib, confirmed by mRFP-GFP-LC3 transfection and LC3 analysis. The mammalian target of rapamycin and a panel of downstream proteins responded to Celecoxib and propelled autophagy machinery to stabilize homeostasis. Ultimately, inhibition of autophagy by silencing autophagy protein 5 disrupted the protective effects of Celecoxib, culminating in apoptosis.

**Conclusion:**

In summary, we have demonstrated a new use for the old drug Celecoxib that treats intervertebral disc degeneration by enhancing autophagy in nucleus pulposus cells and opening a door for treating other degenerative diseases.

**Supplementary Information:**

The online version contains supplementary material available at 10.1186/s40360-022-00633-y.

## Background

Intervertebral disc degeneration (IDD) is a condition affecting a vast population in aging societies globally. It is estimated to affect more than 40% of the population in the United States and causes 20–33% of patients to be unable to work [1], which costs about $100 billion every year due to productivity loss [2]. More importantly, IDD is closely related to other spinal diseases and contributes to low back pain [3].

Intervertebral discs as joints not only provide flexibility and mobility but also act as a cushion to bear bodyweight and withstand forces from outside of the body. Being able to absorb pressure, intervertebral discs are made of gel-like material, which are the extracellular matrix (ECM) and nucleus pulposus cells (NPCs), and exchange substances only through capillaries in endplates. Aggrecan is the major component of proteoglycan, which comprises the ECM, and possesses negatively charged groups with the ability to retain water [4]. These physiological features underscore the nucleus pulposus as a pivot to treating intervertebral disc degneration. What’s more, understanding the mechanism by which NPCs respond to pathological circumstances is key to the treatment of IDD.

Normally, water-rich elastic nucleus pulposus remain unaffected by pressure under normal conditions. However, there are various factors that contribute to IDD, in which inflammation plays a major role and leads to cell death [5, 6]. A research has found that pro-inflammatory cytokine interleukin 1β (IL-1β) expressed higher in degenerative intervertebral discs, and the expression of other members of the IL-1 family also alters in degenerative discs [7]. Gorth, et al. even considered IL-1β, known as a major inflammatory cytokine, a hallmark of IDD [8]. IL-1β induces more cyclooxygenase-2 (COX-2) expression, thus forming a vicious cycle and exacerbates disc degeneration [9]. In patients with severe IDD and osteoarthritis, more than 20-fold of COX-2 expresses in cartilage, and subsequent prostaglandin E2 (PGE_2_) production decreased proteoglycan synthesis [10]. In addition, IL-1β and other inflammatory cytokines stimulate reactive oxygen species (ROS) production, and COX-2 also generates ROS in the course of arachidonic peroxidation [11, 12]. Many studies have found the links between excessive ROS and disruption of biological processes, such as protein misfolding, malfunction of transporters on the cell membrane, DNA damage [13], which further led to apoptosis [14]. Such oxidative stress and biological effects contribute to the development of IDD.

So far, few agreements have been made on the treatment of IDD, and the treatment is usually surgery to relieve symtoms. However, surgery is not a definitive treatment. Besides, the financial burden, and the complications should also be taken into account. Moreover, surgery is not a preventive measure at the early stage of IDD. In a few reports, encountering inflammation by corticosteroids in treating degeneration was not effective and conclusive [15]. Remarkably, non-steroidal anti-inflammatory drugs are promising in treating IDD. Vaudreuil et al. successfully delayed IDD progression caused by puncture with indomethacin [15, 16]. In addition, Su et al. treated porcine chondrocytes with Celecoxib (CXB) and found it enriched ECM components [17]. CXB has been widely used in treating inflammatory diseases due to selectively inhibiting COX-2, thus have fewer side effects than COX-1 inhibitors and corticosteroids while relieving pain [18, 19]. These findings point to selective COX-2 inhibitors as a novel approach. Nevertheless, whether and how CXB postpones the progression of IDD have not been studied.

Autophagy has gained attention as it is implicated in a plethora of diseases, such as autoimmune disease, neurodegenerative disease, and cancer [20]. Autophagosome stabilizes the intracellular environment by degrading dysfunctional organelles and misfolded proteins in response to stimuli [21]. The importance of normal autophagy and the related proteins were found in other disease models, as the impaired autophagy resulted from autophagy protein 5 (Atg5) knockdown lead to protein aggregates and caused neurodegeneration [22]. Altogether, CXB could be a promising agent and autophagy should be the key to IDD treatment.

In this study, we investigated the pathology of IDD from several aspects, including ECM synthesis, ROS production, autophagy, and apoptosis. We hypothesize that CXB increases ECM components and reduces ROS production caused by inflammation. CXB also enhances autophagy via the mammalian target of the rapamycin (mTOR) pathway, thus preventing apoptosis.

## Materials and methods

### NPCs isolation and culture

The process of primary cell isolation was verified by the Animal Care and Use Committee of Fudan University (Research Ethics Committee Reference Number: 2020-023). NPCs were harvested according to the protocol from Bratsman et al. [23]. Briefly, 8-week old Sprague-Dawley rats were euthanized with an intraperitoneal injection of pentobarbital. Rat vertebrae were dissected, and muscles were scraped off until intervertebral discs were exposed. After cutting open intervertebral discs, nucleus pulposus tissues surrounded by annulus fibrosis were isolated with tweezers and digested with 0.25% collagenase P for at least 4 h. Primary cells in tissue mass were planted with high glucose DMEM containing 10% FBS and cultured in an incubator at 37˚C, 1% O_2_. Once cell culture reached 80% of confluency, passaging was performed, and passages one to three were used for the following experiments.

### Cytotoxicity assay

For the exclusion of cell toxicity originating from CXB, a cytotoxicity assay was performed in advance to determine proper working concentration. NPCs were seeded in a 96-well plate and treated with agents on the next day for 24 h. Cell Counting Kit (Yeason) was used and optical density (OD) was measured with a plate reader (FlexStation).

### Western blot

Grew in 6-well plates at a density of 5 × 10^5^, NPCs were subjected to treatments of IL-1β (recombinant from human biological source, SRP3083, Sigma) or CXB (aladdin, c129279, approved by the manufacturer for publication). A monolayer of cell culture was treated with RIPA buffer supplemented with PMF and phosphatase inhibitors prior to protein harvest. Cell lysates were collected and denatured with loading buffer (Beyotime). The protein of interest was separated by SDS-PAGE and transferred to the PVDF membrane (Merck Millipore). Following blocking with 5% milk, PVDF membranes were cropped then incubated with primary antibodies separately for each blot at 4˚C overnight. On the second day, PVDF membranes were washed by TBST and incubated with HRP-conjugated secondary antibodies at RT for one hour, then washed again by TBST. ECL Western blot substrate (Epizyme) was added to a PVDF membrane, and the signal was detected by Clinx ChemiCapture 6000. Protein expressions were semi-quantified by ImageJ 1.53c, which is free & open-source software and can be downloaded at https://imagej.nih.gov/ij/. The protein of interest amount was normalized to GAPDH for analysis [24]. The relative amount of LC3-I and LC3-II serves as an indicator for autophagic flux, and Bafilomycin A1 (Baf A1) was applied to inhibit autolysosome formation for unmasking faster LC3-II degradation with autophagy [25]. All antibodies were applied at a final concentration of 1/1000. More information about antibodies used are as follows: β-Actin (AF5003), Bcl-2 (AF6285), LC3B (AL221), Beclin-1 (AF5123) from Beyotime. HRP-conjugated Affinipure Goat Anti-Rabbit IgG (H + L) (Proteintech Cat# SA00001-2, RRID:AB_2722564), GAPDH (Proteintech Cat# 10494-1-AP, RRID:AB_2263076), Collagen Type I(Proteintech Cat# 14695-1-AP, RRID:AB_2082037), Collagen Type II (Proteintech Cat# 28459-1-AP, RRID:AB_2881147), Aggrecan (Proteintech Cat# 13880-1-AP, RRID:AB_2722780), ATG5 (Proteintech Cat# 66744-1-Ig, RRID:AB_2882092). Phospho-mTOR (Cell Signaling Technology Cat# 2971, RRID:AB_330970), mTOR (Cell Signaling Technology Cat# 2983, RRID:AB_2105622), Phospho-Akt (Cell Signaling Technology Cat# 4060, RRID:AB_2315049), Akt (pan) (Cell Signaling Technology Cat# 4691, RRID:AB_915783), Phspho-S6 Ribosomal Protein (Cell Signaling Technology Cat# 4858, RRID:AB_916156), S6 Ribosomal Protein (Cell Signaling Technology Cat# 2217, RRID:AB_331355), Phospho-4E-BP1 (Cell Signaling Technology Cat# 2855, RRID:AB_560835), 4E-BP1 (Cell Signaling Technology Cat# 9452, RRID:AB_331692), Caspase-9 (Cell Signaling Technology Cat# 9508, RRID:AB_2068620), Bax (Cell Signaling Technology Cat# 5023, RRID:AB_10557411).

### Intracellular and mitochondrial ROS assay by Microscopy and Flow Cytometry

After removal of culture medium and washing with PBS, NPCs were labeled with DCFH-DA (Sigma) and incubated at 37˚C, which is oxidized to DCF intracellularly and emits fluorescence. Morphology and intensity of fluorescence correspond to hydroxyl, peroxyl, and other reactive oxygen species activity in cells. Images of cells were taken by Olympus DP74. The technique for staining intracellular ROS for cytometry analysis is the same as fluorescence microscopy. After labeling, NPCs were trypsinized and resuspended in Falcon round-bottom tubes at a density of 3 × 10^6^. Ten thousand events were collected for each sample, and the mean fluorescence intensity (MFI) was calculated by Flowjo X for further analysis.

### siRNA transfection

NPCs were seeded in a 6-well plate the day before transfection. On the next day, Lipofectamine 3000 transfection reagent (Thermofisher) and Opti-MEM Reduced Serum Medium (Thermofisher) were used following the manufacturer’s protocol. NPCs were transfected with autophagy protein 5 (Atg5) siRNA purchased from RiboBio company at a concentration of 50nM or negative control for 24 h. After transfection, NPCs were subjected to different treatments for further analysis. The efficiency of siRNA interference and exclusion of off-target effects were confirmed with one scrambled and two targeting sequences by western blot [26].

### Autophagy tandem sensor mRFP-GFP-LC3 transfection

NPCs were seeded in a 24-well plate at a density of 3 × 10^5^ the day before transfection. When confluency reached 50%, Autophagy Tandem Sensor mRFP-GFP-GFP (HANBIO) were added to each well at 50 multiplicity of infection (MOI), which was optimized by titration. After transfection, NPCs were subjected to different treatments for 24 h. Green/red puncta were viewed, and images were captured by Olympus DP72. Images were analyzed and merged with ImageJ 1.53c.

### YO-PRO-1/PI apoptosis assay

NPCs were seeded in 12-well plates and subjected to the indicated treatments. The culture medium was removed and stained with YO-PRO-1 and Propidium iodide (Beyotime) at 37˚C for 30 min. Staining buffer was removed, then NPCs were washed with PBS twice, disassociated with trypsin-EDTA, then collected in round-bottom tubes. Next, NPCs were centrifuged and washed, then resuspended in PBS with 5% FBS. Ten thousand events were recorded for each sample, and data were analyzed by Flowjo X.

### Statistical analysis

All the experiments were performed at least three times. The data were presented as mean with standard deviation. Graphs were made by GraphPad Prism 9. The symmetry and shape of histogram and boxplot and Shapiro-Wilk test were used to assess the normal distribution. Statistical significance was determined by either Student’s t-test or ANOVA with Tukey-Kramer’s post hoc test. Statistical analysis was performed with SPSS 28.0.1. The P-value less than 0.05 was considered significant.

## Results

### CXB restored ECM synthesis suppressed by IL-1β in dosage and timely manner

**Fig. 1:**
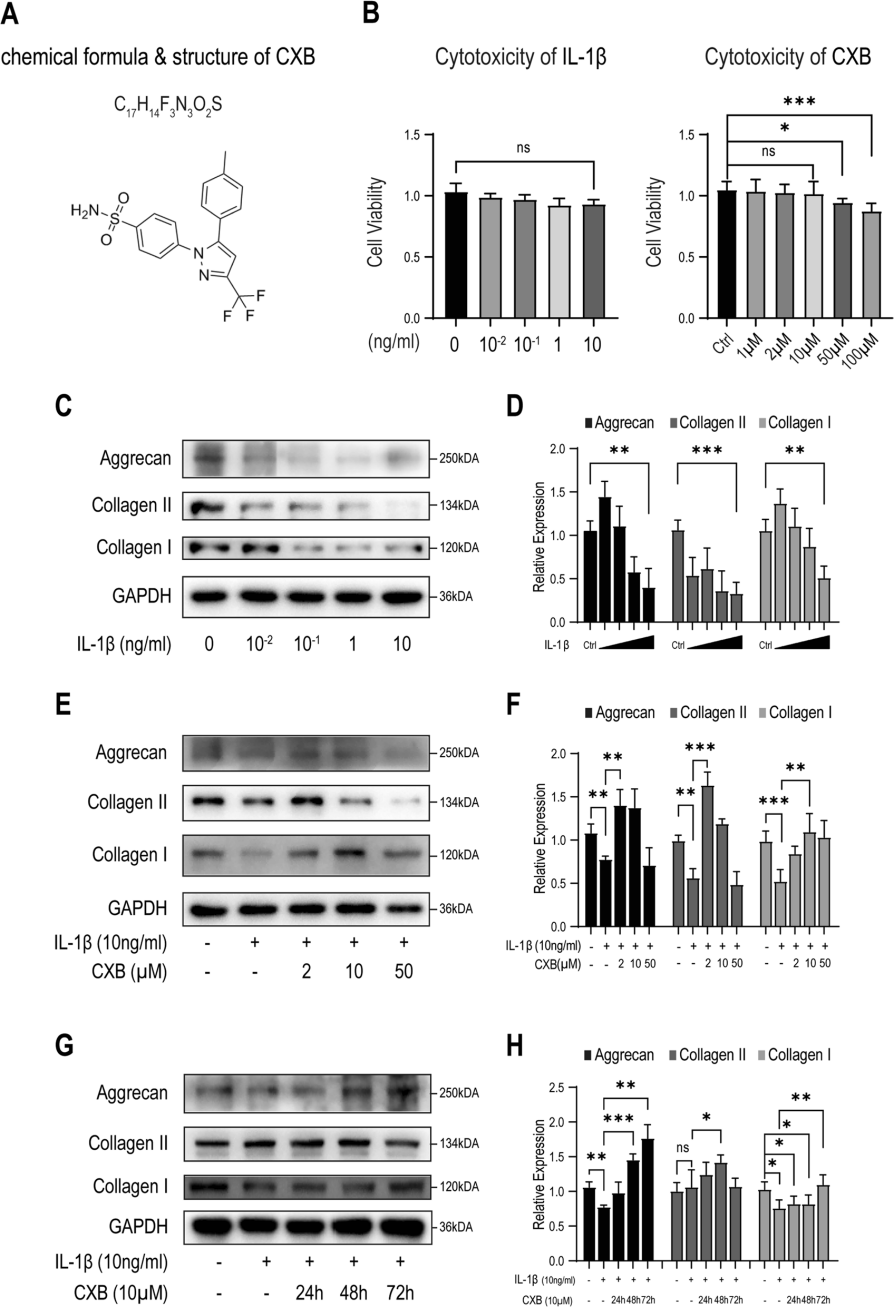
IL-1β inhibited ECM synthesis and was restored by CXB in a dose and time-dependent manner in NPCs. **A** Chemical formula and structure of CXB provided by the manufacturer. **B** Cytotoxicity assay of IL-1β and CXB. **C**, **D** Expression of aggrecan, type I and II collagen decreased as the concentration of IL-1β increased. **E**, **F** Treating with CXB for 24 h restored ECM synthesis in a dose-dependent manner. **G**, **H** NPCs pre-treated with 10 µM CXB for 24, 48, 72 h, and treated with IL-1β for 24 h enhanced ECM synthesis in a time-dependent manner. The grouping of blots were cropped from different parts of different blots. Data are represented as mean with SD of more than three repeats, performed in triplicates. *P ≤ 0.05, **P ≤ 0.01, *** *P* ≤ 0.001

Before studying the effects of IL-1β and CXB (Fig. 1 A) on ECM synthesis, we screened various concentrations for proper ones (Fig. 1B). The cytotoxicity of IL-1β was non-significant below 10 (ng/ml). The cytotoxicity of CXB increased slightly at higher concentrations, but it was bearable to NPCs at lower concentrations. Increasing IL-1β concentrations were associated with a reduction in aggrecan, type I and II collagen, which are major components of ECM. At 10 (ng/ml) of IL-1β, ECM decreased by more than 50% (Fig. 1 C, D). Next, we counteracted inflammation by applying various concentrations of CXB combined with 10 ng/ml of IL-1β to see how it affected protein synthesis. The adverse effects on NPCs were relieved, as aggrecan, type I and II collagen expressions improved (Fig. 1E, F). The effects on NPCs were studied by treating them for 24 to 72 h with 10 µM of CXB. As the treatment duration increased, ECM synthesized by NPCs also increased (Fig. 1G, H). We found that type II collagen was affected more by IL-1β stimulation and CXB intervention. However, the ECM synthesis returned to a low level if the concentration of CXB was way too high.

### Intracellular and mitochondrial ROS generation was induced by IL-1β and suppressed by CXB

**Fig. 2:**
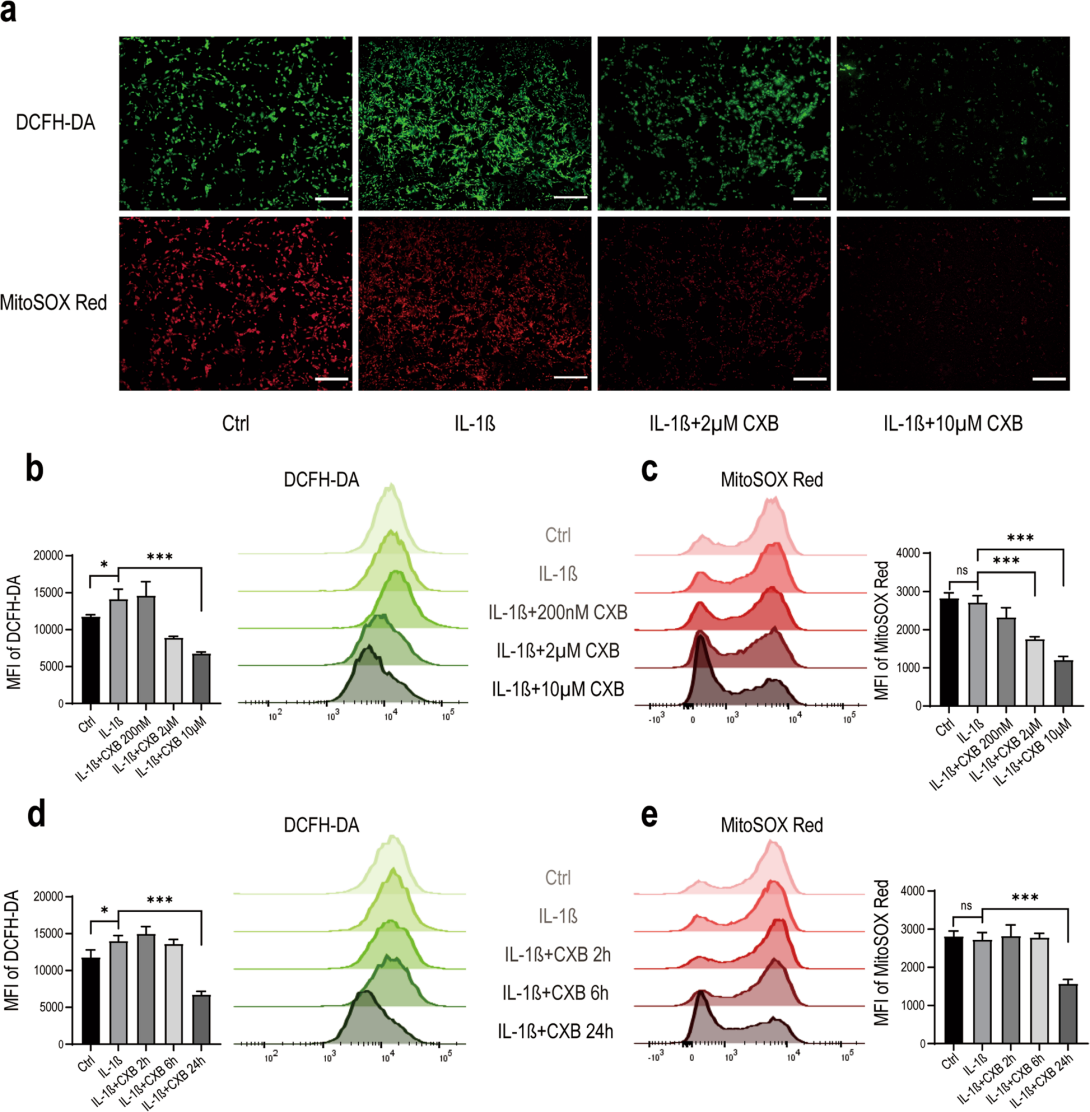
CXB reduced intracellular ROS and mitochondrial ROS production in NPCs. **A** Representative images of NPCs treated with IL-1β alone or with 2 µM or 10 µM CXB for 24 h and stained for intracellular ROS and mitochondrial ROS by DCFH-DA and MitoSOX Red at 37˚C, 30 min. **B**, **C** CXB reduced intracellular ROS and mitochondrial ROS production at higher concentrations. **D**, **E** 10 µM CXB reduced intracellular ROS and mitochondrial ROS production significantly at 24 h. Flow cytometry analysis was calculated as mean fluorescence intensity (MFI). Data were represented as mean with SD. Scale Bars, 200 μm. *P ≤ 0.05, **P ≤ 0.01, *** *P* ≤ 0.001

To distinguish the source and composition of ROS, we chose DCFH-DA as it is transformed by esterase and reacts with a variety of ROS. We also utilized a novel probe MitoSOX Red which only detects superoxide in mitochondria [27]. After different treatments of IL-1β and CXB, we examined intracellular and mitochondrial oxidative stress simultaneously by DCFH-DA and MitoSOX Red. As CXB was added to IL-1β treated NPCs, the green fluorescence brightness decreased, as did the red fluorescence produced by MitoSOX Red (Fig. 2 A). Flow cytometry was used to find out whether CXB alleviated oxidative stress by measuring the intensity of more cells at once. The results of flow cytometry for treating NPCs with different concentrations of CXB were consistent with fluorescence microscopy (Fig. 2B, C). Since ROS balance is dynamic progress rather than a fixed value, we treated NPCs with CXB for different periods. Interestingly, CXB demonstrated ROS suppressing effect that reached its peak at 24 h (Fig. 2D, E). These results showed that IL-1β caused abnormal electron transport as more ROS were created, and it was relieved by the anti-inflammatory effect of CXB.

### Autophagosome formation was up-regulated by CXB evidenced by mRFP-GFP-LC3 and western blot

**Fig. 3:**
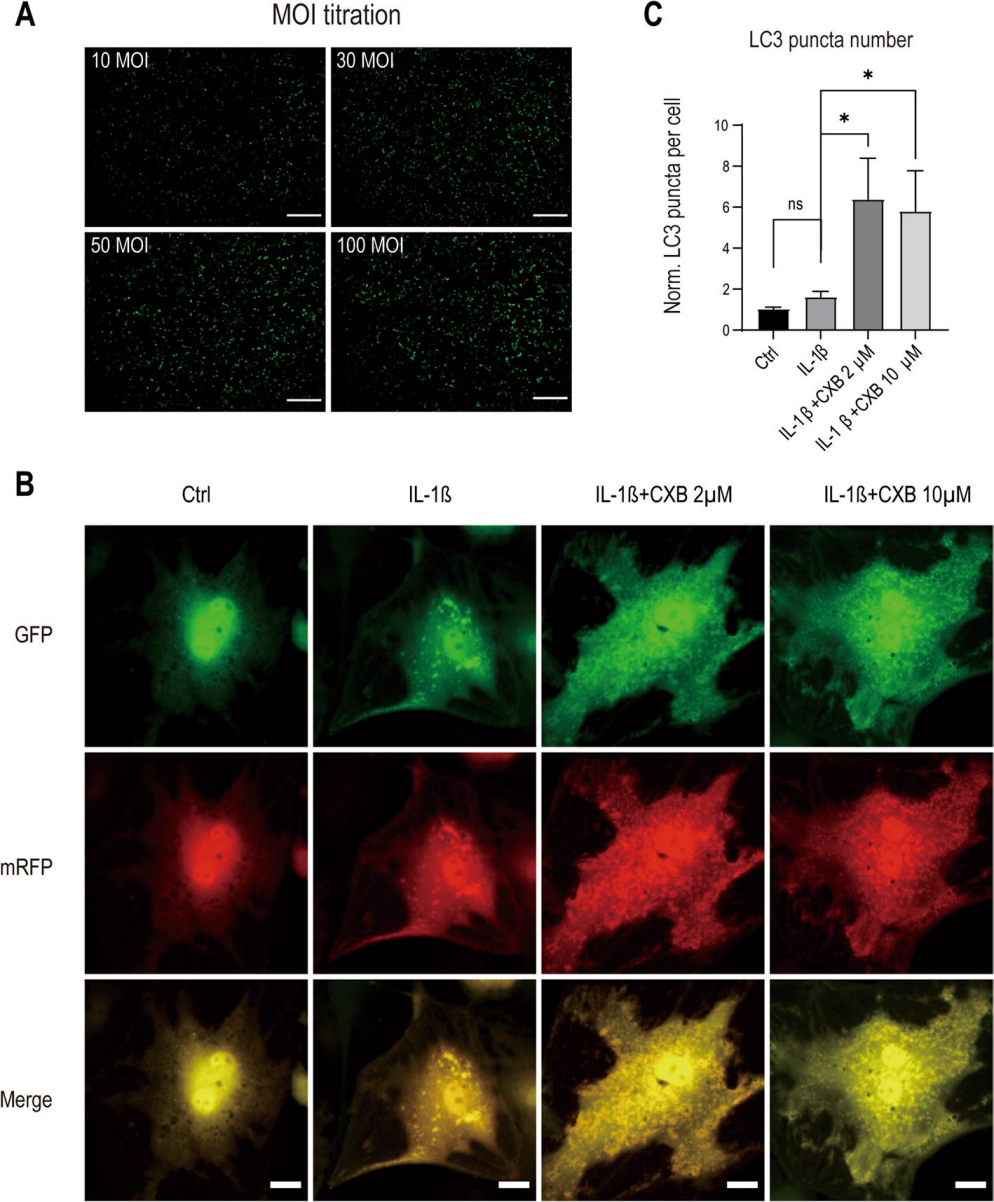
CXB enhanced autophagy in NPCs transfected with mRFP-GFP-LC3. **A** MOI titration assay for the optimal transfection. **B** Representative images and numbers of puncta of NPCs transfected with mRFP-GFP-LC3 for 6 h and replaced with fresh culture medium and subjected to IL-1β alone or with 2 µM or 10 µM CXB. Yellow puncta indicate autophagosome formation and were quantified (**C**). Each experiment was repeated three times. Scale bars for MOI titration, 200 μm. Scale Bars for autophagy, 30 μm

**Fig. 4:**
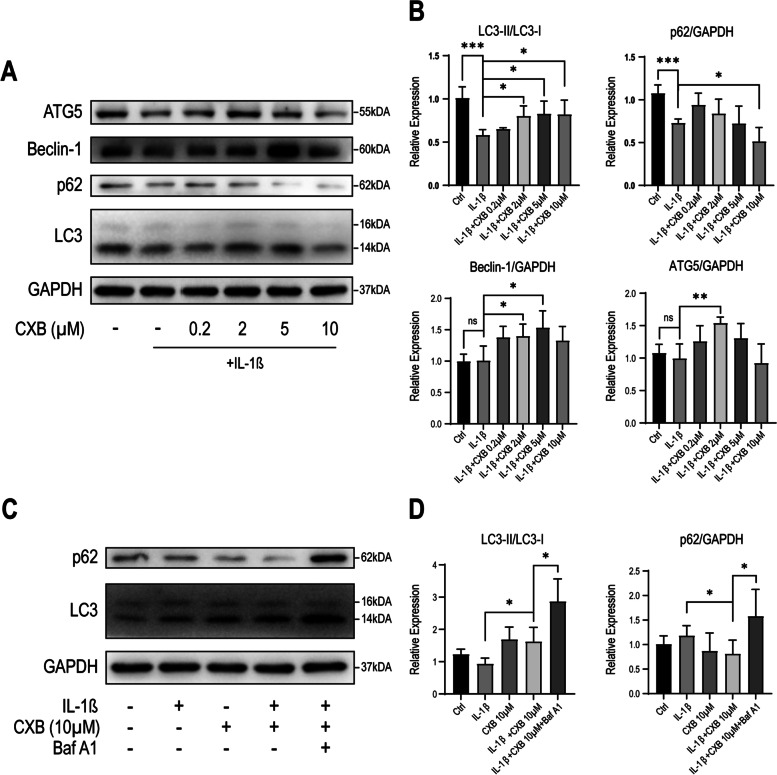
CXB enhanced autophagy in a dose-dependent manner. **A**, **B** NPCs were treated with IL-1β alone or with 0.2, 2, 5, 10 µM of CXB and analyzed with LC3-II/LC3-I, p62, ATG5, and Beclin-1. **C**, **D** NPCs were treated with IL-1β alone or with 10 µM CXB. Furthermore, 200nM Baf A1 was added to confirm autophagosome enhancement by inhibiting autophagosome formation. The grouping of blots were cropped from different parts of different blots. Data were represented as mean with SD. *P ≤ 0.05, **P ≤ 0.01, *** *P* ≤ 0.001

Given that protein synthesis and oxidative stress have changed in inflamed NPCs, we reasoned that autophagy plays a major role in homeostasis and counteracts inflammation. To monitor autophagy formation in real-time, we transfected NPCs with Autophagy Sensor mRFP-GFP-LC3. Optimal MOI for transfection was verified by titration (Fig. 3 A). Compared to the control group, yellow puncta in the IL-1β group increased slightly, indicating that autophagosome formed more when treated with IL-1β. However, yellow puncta grew tremendously when concentrations of CXB treated NPCs with IL-1β (Fig. 3B) and quantified (Fig. 3 C). Autophagy requires a few experiments to validate and complement each other’s results. To do so, we analyzed autophagic flux by measuring LC3-I and LC3-II. When NPCs were being treated with increasing concentrations of CXB, the ratio of LC3-II to LC3-I also rised, indicating enhancement of autophagic flux under IL-1β. Opposing to LC3, p62 decreases by autophagic degradation (Fig. 4 A, B). Furthermore, we compared autophagic flux between samples by inhibiting the fusion of autophagosome and lysosome with Bafilomycin A1 (Fig. 4 C, D) [28]. LC3-II to LC3-I ratio increased in NPCs treated with CXB combining IL-1β, and Baf A1 blocked the autophagic degradation, evidenced by even more of LC3-II.

### mTOR signaling pathway facilitated autophagy in response to CXB

**Fig. 5:**
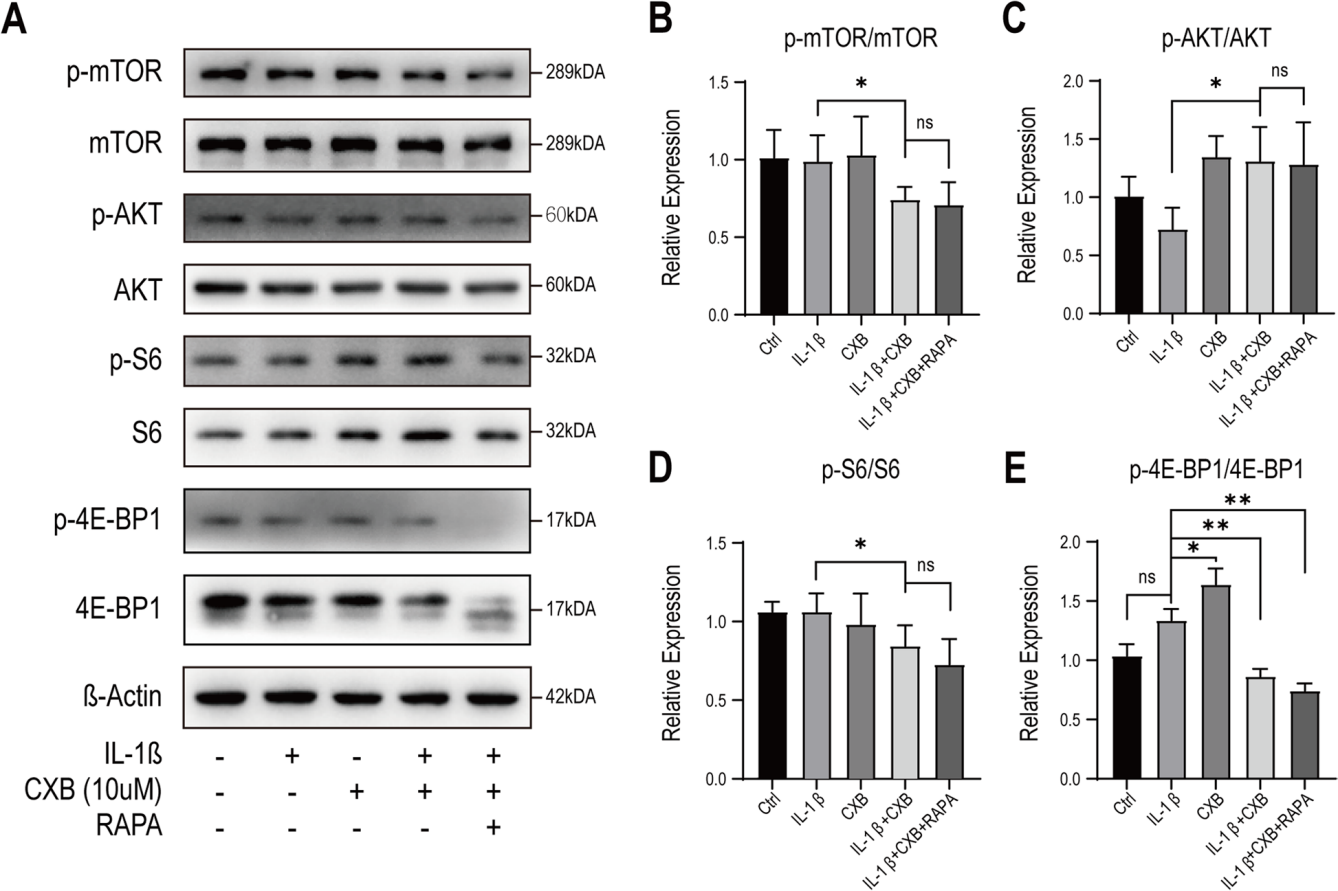
CXB inhibited mTOR signaling pathway and downstream molecules. **A**-**E** NPCs were treated with IL-1β alone or with 10 µM CXB. 200 nM RAPA was added in addition to IL-1β and CXB as a positive control. Phosphorylation of mTOR, Akt, rpS6, and 4E-BP1 were analyzed for activity relating to autophagy, cell growth, and mRNA translation. The grouping of blots were cropped from different parts of different blots. Data were represented as mean with SD. *P ≤ 0.05, **P ≤ 0.01, *** *P* ≤ 0.001

In search of the mechanism that CXB regained ECM synthesis and reduced ROS production by autophagy, we focused on kinases. In response to environmental cues, such as starvation and stimuli, the mTOR pathway regulates cell growth and survival, which has become our primary concern [29]. Therefore, we sought to clarify how CXB affects mTOR. This was done by using RAPA to examine mTOR and downstream molecules’ activity relating to survival and apoptosis. We performed western blot and analyzed multiple downstream proteins of mTOR signaling to investigate how they work together to promote autophagy and other functions. We found that CXB inhibited mTOR when NPCs were stimulated by IL-1β, and this effect was confirmed by Rapamycin (RAPA) (Fig. 5 A, B). In the contrast, protein kinase B (Akt) was upregulated and unchanged by RAPA (Fig. 5 C). The downstream ribosomal protein S6 (rpS6) was also downregulated to a greater degree (Fig. 5D). 4E-binding protein-1 (4E-BP1) is another downstream element of mTOR. In eukaryotic cells, 4E-BP1 binds to eukaryotic initiation factor 4E (eIF4E) that initiates mRNA translation. In response to mTOR, 4E-BP1 (Fig. 5E) was dephosphorylated, thus associated with eIF4E and inhibited cap-dependent translation [30]. These results showed that mTOR activity and its downstream effectors, mediated autophagy which was induced by CXB.

### Autophagy prevents apoptosis and inhibition of ATG5 lead to apoptosis

**Fig. 6:**
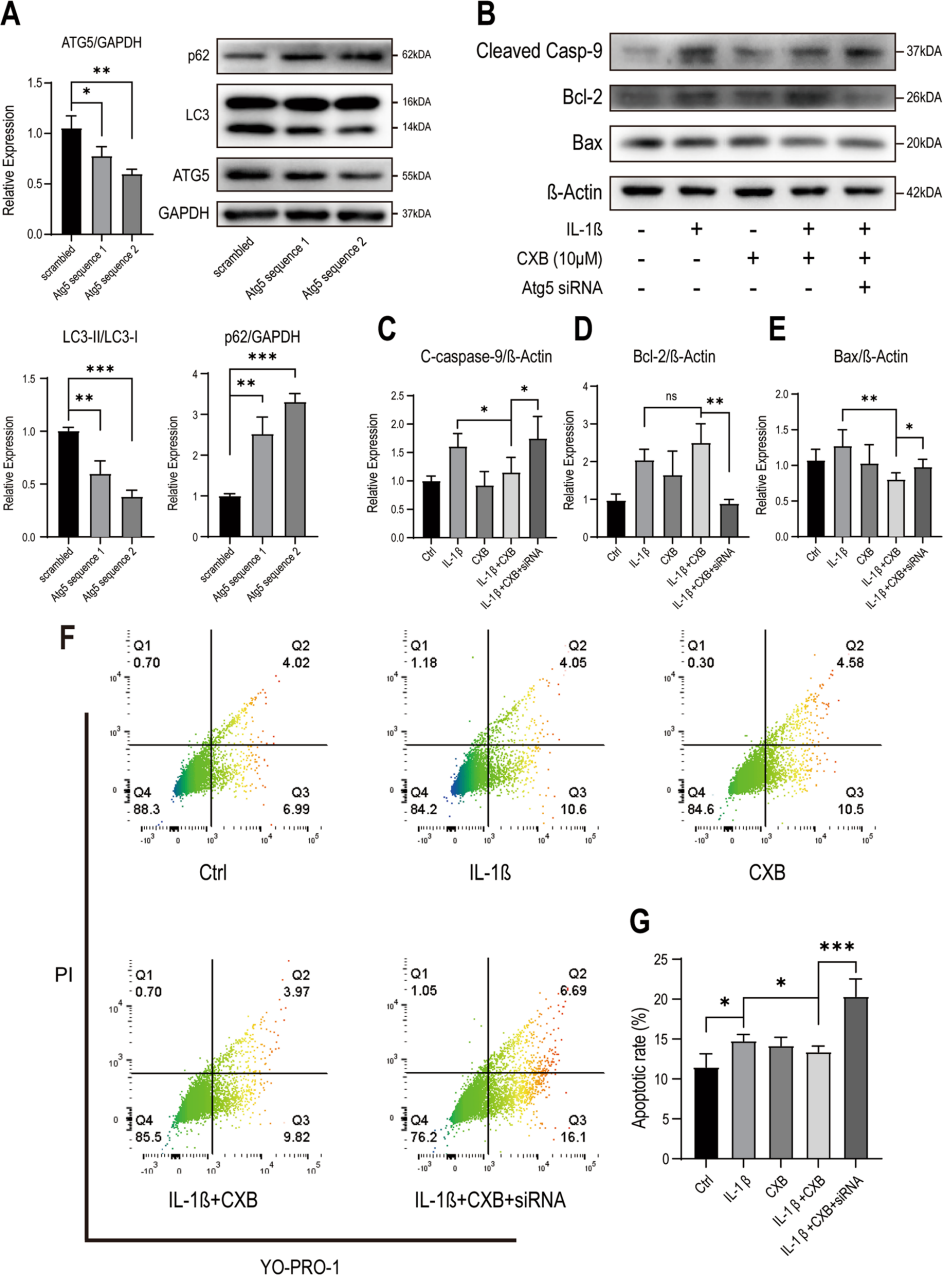
CXB has protective effects on IL-1β treated NPCs, and inhibition of autophagy leads to apoptosis. **A** Atg5 silencing and autophagy inhibition was evaluated by two different sequences of siRNA. **B**-**E** NPCs were treated with IL-1β, 10 µM CXB, or with inhibited autophagy. Pro-survival protein Bcl-2 and pro-apoptotic protein cleaved caspase-9, Bax were analyzed by Western blot. The grouping of blots were cropped from different parts of different blots. **F**, **G** For apoptosis analysis, NPCs were treated with IL-1β, 10 µM CXB, or inhibited with autophagy, then stained with YO-PRO-1/PI for FACS. Values in dot plots were represented in percentage. Data are represented as mean with SD of three repeats. *P ≤ 0.05, **P ≤ 0.01, *** *P* ≤ 0.001

Concerning findings in autophagy, we examined if CXB treatment upon autophagy exhibits anti-apoptotic and pro-survival effects by Atg5 siRNA silencing. Firstly, NPCs were transfected with Atg5 siRNA at 50 nM for 24 h before subjecting to IL-1β and CXB. Knockdown of Atg5 translation to 40% was verified by western blot (Fig. 6 A). Atg5 silencing solely doesn’t lead to apoptosis of nucleus pulposus cells [26, 31]. After 24 h of indicated treatment, cleaved caspase-9, B-cell lymphoma 2 (Bcl-2), BCL2 Associated X (Bax) were analyzed by western blot. In NPCs treated with IL-1β alone, pro-apoptotic protein cleaved caspase-9 and Bax were relatively higher than NPCs treated with 10 µM combining IL-1β. On the contrary, pro-survival protein Bcl-2 was the highest in NPCs treated with CXB and IL-1β among the others (Fig. 6B-E). Apoptosis was monitored in NPCs by YO-PRO-1 and PI as disruption of the cell membrane increased their diffusion. As shown in (Fig. 6 F, G), the differences between the treatment with IL-1β or CXB were not significant. However, inhibition of autophagy in NPCs lead to significant apoptosis. These results suggest the anti-apoptotic effect of CXB by autophagy enhancement. Furthermore, inhibition of autophagy by Atg5 lead to significant apoptosis.

## Discussion

Our study found that IL-1β, as an inflammatory cytokine, affected ECM synthesis and ROS production, which contributed to NPC degeneration. CXB reversed the deleterious effect of IL-1β by modulating autophagy. The mTOR signaling pathway was the principal regulator upon activating autophagy and maintained NPCs homeostasis. Autophagy was found to be a critical role in promoting cell survival and viability by reducing apoptotic proteins and retaining cell membrane integrity.

We noticed that major ECM proteins, which correlates to NPCs function and water retention, were reduced by IL-1β stimulation. It was proposed that overactive matrix metalloproteinases (MMP) induced by IL-1β lead to the structural change of intervertebral discs [26]. Furthermore, IL-1β promotes blood vessel and nerve ingrowth, followed by leukocyte infiltration and pain (35). In addition to IL-1β, other inflammatory cytokines including IL-6, IL-17, and TNF were also characterized in IDD. The much higher expression of IL-1β among the others justifies its usage as a stimulator and the results that came out are representative (33, 34). At low and medium concentrations with a longer duration of treatment, CXB regained protein synthesis by NPCs, suggesting an overall positive effect. Mastbergen et al. found that CXB enhanced proteoglycan synthesis rather than the nonselective cyclooxygenase inhibitor Indomethacin in arthritic human cartilage culture, supporting ECM production is benefited by selectively inhibiting COX-2 [32].

We found that intracellular ROS increased slightly at a lower CXB concentration and decreased significantly at higher CXB concentrations. Unexpectedly, CXB takes a considerable amount of time to reduce ROS levels. Unlike intracellular ROS, mitochondrial ROS decreased more at every higher CXB concentration.

IL-1β and CXB might change ROS catalysis of the superoxide dismutase (SOD) to a different exent in the cytosol and mitochondira, and result in a discrepancy between consequent intracellular and mitochondrial ROS [33, 34]. It was found that CXB affects ROS production variably in several studies, and altered mitochondrial function in a different context should be responsible for the difference [35, 36]. Mitochondria are the primary sources of ROS, while pathological factors such as inflammation and hypoxia affect complex II of the mitochondrial respiratory chain and NADPH oxidase (NOX) activity. In accelerated aging mice, mitochondria-derived ROS was identified to reduce proteoglycan synthesis [37]. Since CXB reduced mitochondrial ROS, it has the potential in treating ROS-related diseases [38, 39].

Autophagy was characterized in the progression of IDD, and the expression of autophagy-related genes are responsive to IL-1β stimulation [40]. CXB was proven to activate autophagy in several cancer cell lines and hepatocytes [41, 42]. In our study, CXB induced numerous autophagic puncta in NPCs, and LC3-II to LC3-I ratio increased consecutively with every higher CXB concentration, suggesting autophagy of NPCs responded well to CXB. What’s more, misinterpretation of LC3 analysis was ruled out by blocking autophagosome-lysosome fusion, which degrades LC3-II. Other alternative autophagy indicators, including ATG5 and Beclin-1, were also compatible with LC3 analysis, indicating canonical pathway of autophagy [43]. Concentrations of CXB for inducing autophagy are different in several studies. In our study, we used lower concentrations to induce autophagy, as higher concentrations are not protective in terms of ECM production. In contrast to elevated expression of COX-2 and autophagy in NPCs, cancer cells are less sensitive to CXB without IL-1β stimulation in other studies, [41, 42]. Since CXB pertaining to autophagy in IDD has not been studied to date, our work described the interplay between CXB and autophagy for the first time.

The mTOR detects environmental stimuli and regulates autophagy by UNC-51-like kinase 1 (ULK1) and Beclin-1. It also regulates protein synthesis by rpS6 and cap-dependent translation by 4E-BP1 [30, 44]. In our study, CXB moderately modulated mTOR activity, followed by the downstream effectors rpS6 and 4E-BP1. In addition to the mTOR phosphorylation [45, 46], other proteins including p53 and Bcl-2 also take part in CXB-induced autophagy [41, 47]. More specifically, mTOR regulated autophagy by forming a protein complex with the regulatory-associated protein of mTOR (RAPTOR) as mammalian target of rapamycin complex 1 (mTORC1). Specifically, selective inhibition of RAPTOR within mTORC1 rather than mTORC2 activates autophagy with protective effects [48]. In addition to CXB taking effects at mTORC1, inhibition of downstream ribosomal protein S6 kinase beta-1 (p70/S6K) by anti-inflammatory drugs A771726 enhances autophagy as well [49]. Moreover, tuberous sclerosis complex (TSC) is another potential target as an upstream regulator of mTORC1 for enhancing autophagy [50]. All the findings revolve around mTORC1 as a promising target for autophagy enhancement therapy under various conditions. However, therapies that aim to modulate protein kinases raise other concerns, as many kinases cowork with other proteins and regulate a plethora of effectors, of which the inhibition leads to side effects. For example, RAPA prevents atherosclerosis by inhibiting mTORC1, it also leads to dyslipidemia by the concurrent inhibition of mTORC2 in the liver [51]. Thus, more specific targets than kinases are of utmost importance for fewer adverse effects while modulating autophagy. Nevertheless, this study is a proof of concept that CXB dephosphorylates mTOR signaling pathway and activates autophagy.

We applied siRNA to inhibit autophagy and excluded the off-target effect, rather than using 3-Methyladenine (3-MA) due to low solubility and interference in normal cell activity [52]. ATG5 participates in autophagosome membrane formation and knockdown of ATG5 further leads to diversified consequences regarding degeneration, autoinflammatory diseases, and compromised immune system in mice [26, 53, 54]. Given its critical role in normal health, ATG5 is an ideal protein for investigating autophagy in apoptosis. CXB protected NPCs from IL-1β stimulation, and without normal autophagy, Bcl-2, cleaved caspase-9, and Bax mediated mitochondrial apoptosis [55]. Interestingly, the interaction of Bcl-2 and Beclin-1 as autophagy regulators have been reported [41, 56]. What role does Bcl-2 play in NPCs’ autophagy induced by CXB needs more experiments to clarify. In addition, cell membrane integrity was also disrupted if autophagy was blocked, in consistent with mitochondrial apoptosis in our study. Not only apoptosis, ATG5 and autophagy block also play an critical role in senescence [31]. Together, autophagy and ATG5 are crucial to apoptosis and other functional characteristics of NPCs and further contribute to the development of IDD.

For the entire study, only rat NPCs were used, so clinical relevance was limited. NPC phenotypes were gradually lost by more passages, although we managed to use early passages. In the end, we found another approach for ameliorating NPCs degeneration in treating IDD rather than simply relieving pain. In hindsight, we look forward to performing in vivo studies and evaluations in the human intervertebral disc.

## Conclusion

The inflammation induced by IL-1β interfered with ECM components, including aggrecan, type I and II collagen. CXB restored ECM components in a dose and timely-dependent manner. CXB also suppressed intracellular and mitochondrial ROS production in a dose and time-dependent manner. CXB significantly enhanced autophagy, which was carried out by modulating the mTOR signaling pathway and downstream effectors, rpS6, and 4E-BP1. The ATG5-mediated autophagy is crucial in preventing apoptosis. In summary, CXB demonstrated protective effects by enhancing autophagy on NPCs. More importantly, autophagy activated by CXB offers a novel strategy in treating other degenerative diseases.

## Supplementary Information

Additional file 1: Supplementary material.

## Data Availability

The datasets used and/or analyzed during the current study are available from the corresponding author on reasonable request.

## Abbreviations

IDD: intervertebral disc degeneration
ECM: extracellular matrix
NPCs: nucleus pulposus cells
IL-1β: interleukin 1β
COX-2: cyclooxygenase-2
Atg5: autophagy protein 5
PGE_2_: prostaglandin E2
CXB: Celecoxib
mTOR: mammalian target of the rapamycin
OD: optical density
Baf A1: Bafilomycin A1
MOI: multiplicity of infection
RAPA: Rapamycin
rpS6: ribosomal protein S6
4E-BP1: 4E-binding protein-1
Akt: protein kinase B
Bcl-2: B-cell lymphoma 2
Bax: BCL2 Associated X
MMP: matrix metalloproteinases
SOD: superoxide dismutase
NOX: NADPH oxidase
ULK1: UNC-51-like kinase 1
mTORC1: mammalian target of rapamycin complex 1
p70/S6K: ribosomal protein S6 kinase beta-1
TSC: tuberous sclerosis complex
3-MA: 3-Methyladenine
