# Supplementary material for: Celecoxib activates autophagy by inhibiting the mTOR signaling pathway and prevents apoptosis in nucleus pulposus cells
Source: BMC Pharmacol Toxicol. 2022 Dec 1;23:90. doi: 10.1186/s40360-022-00633-y (PMC9714067; doi:10.1186/s40360-022-00633-y)
Supplement: Supplementary file 1 — Supplementary material. [file 40360_2022_633_MOESM1_ESM.docx]

The following images are original western blot images in Figures. We crop blots according to molecular weight before probing with primary antibodies. Multiple exposure images are also provided, unfortunately, some multiple exposure images are lost.

**Blots in Figure 1 C**


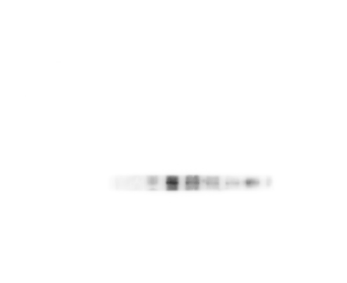

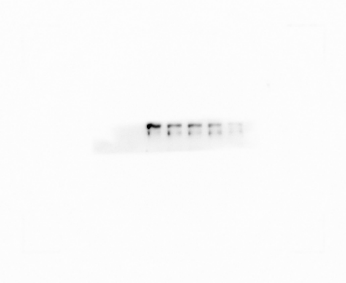


Aggrecan Collagen Ⅱ


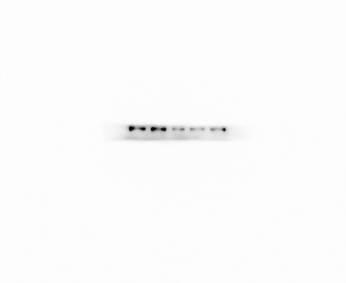

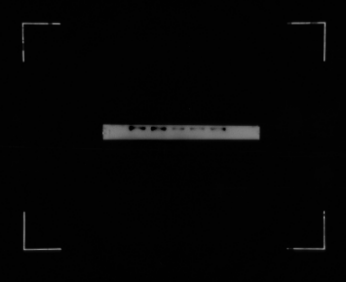


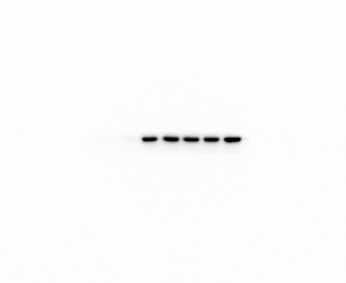


Collagen Ⅰ GAPDH

**Blots in Figure 1 E**


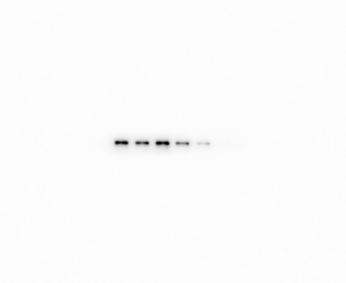

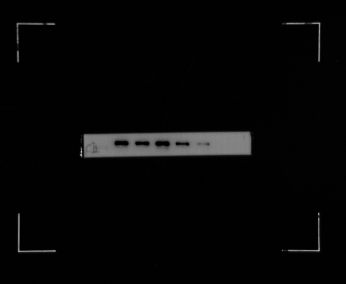

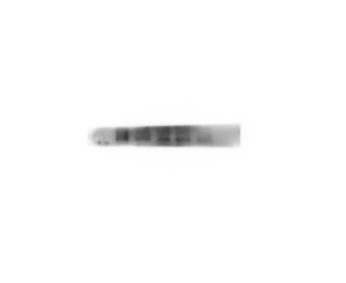


Aggrecan Collagen Ⅱ


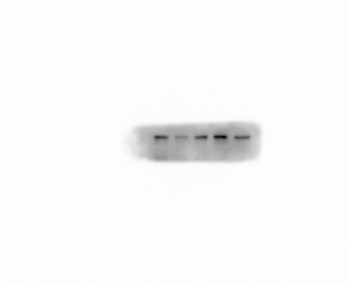

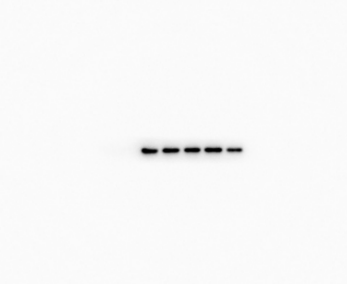


Collagen Ⅰ GAPDH

**Blots in Figure 1 G**


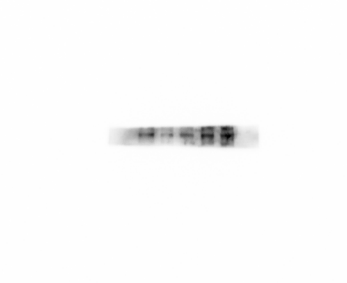

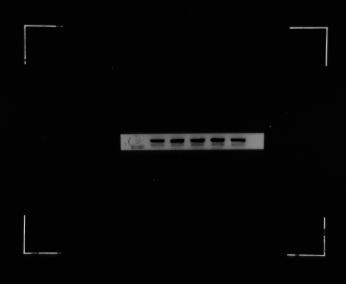

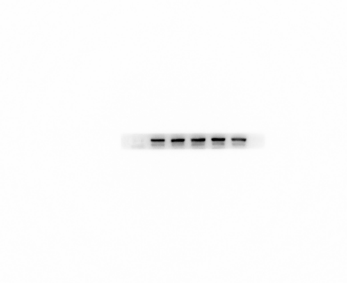


Aggrecan Collagen Ⅱ


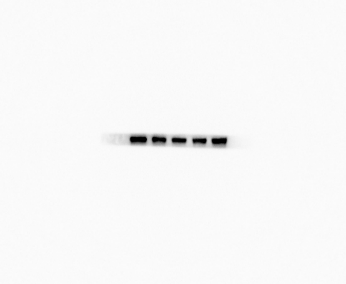

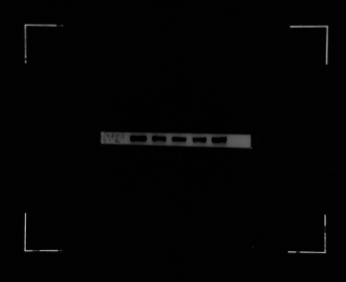

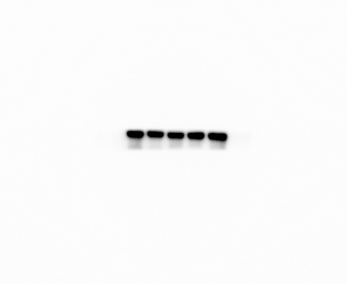


Collagen Ⅰ GAPDH


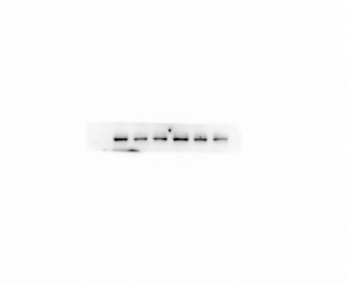

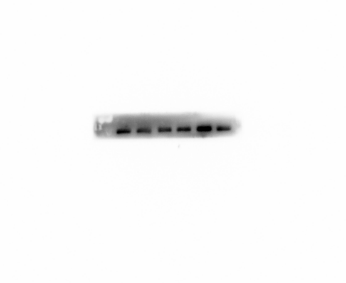

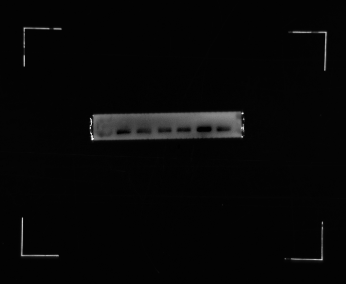

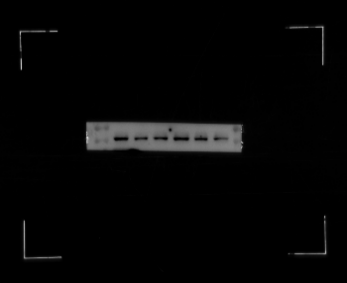
**Blots in Figure 4 A**


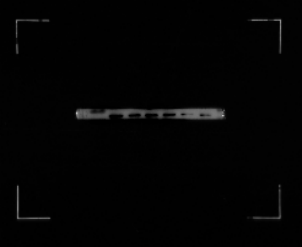

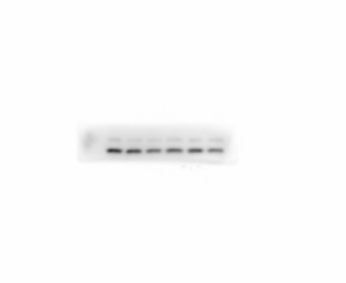

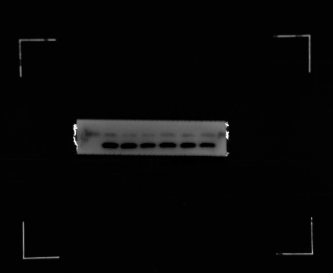

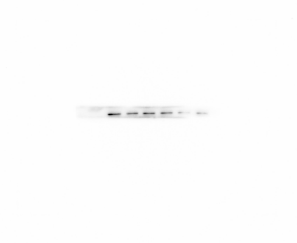
 ATG5 Beclin-1

p62 LC3


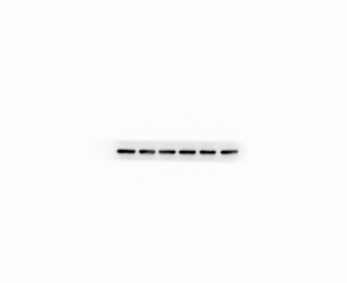

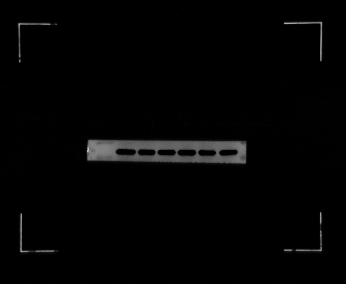


GAPDH


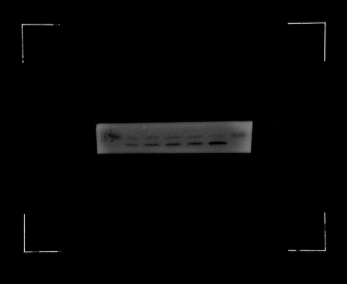

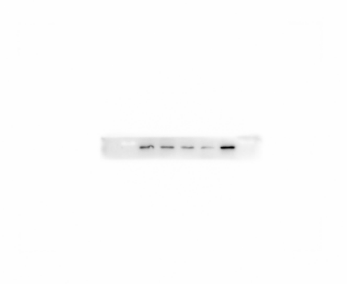

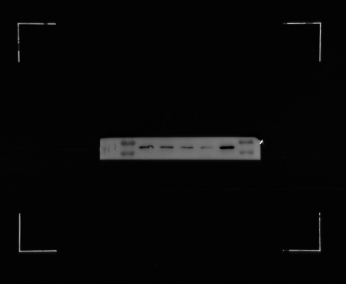
**Blots in Figure 4 C**


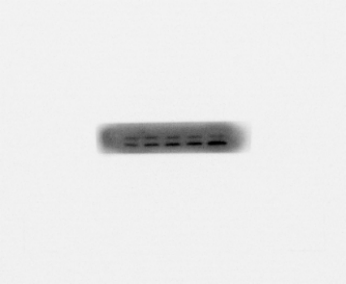
 LC3 p62


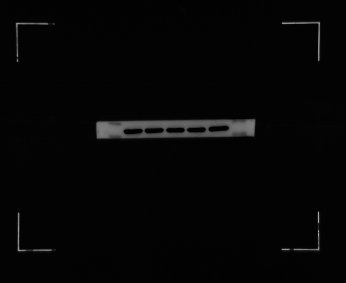

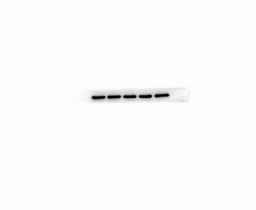


GDPDH

**Blots in Figure 5 A**


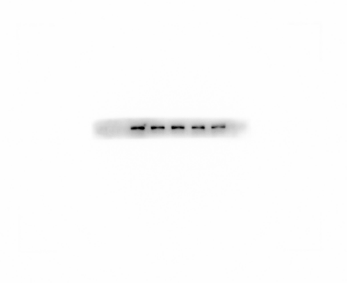

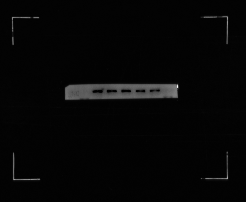

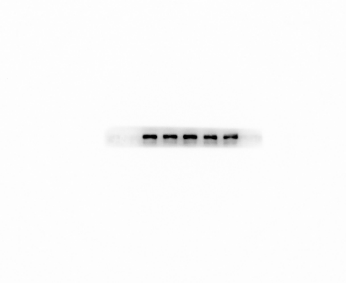


stripping

p-mTOR T-mTOR


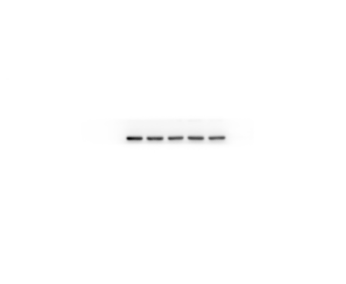

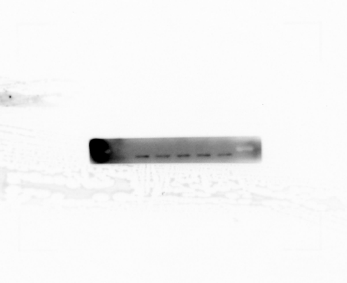

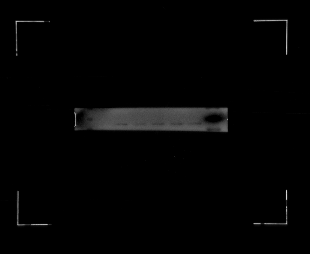


stripping

p-AKT T-AKT


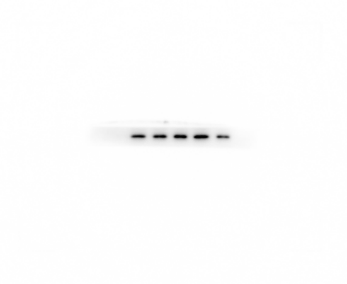

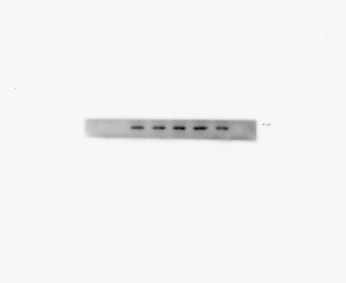

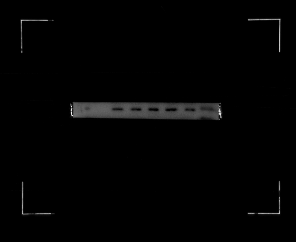


stripping

p-S6 T-S6


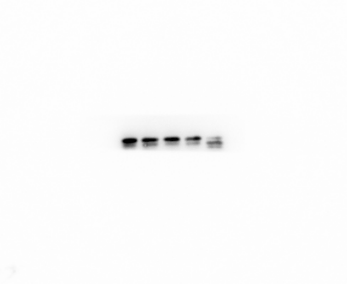

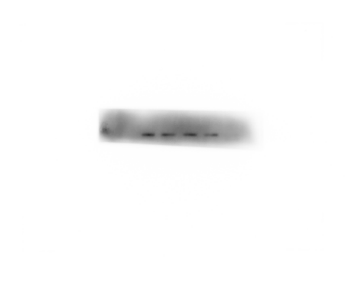

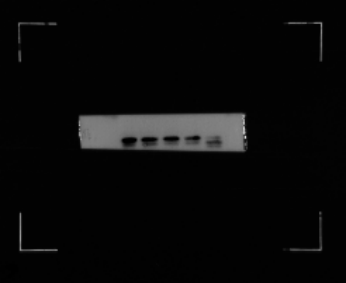


stripping

p-4E-BP1 T-4E-BP1


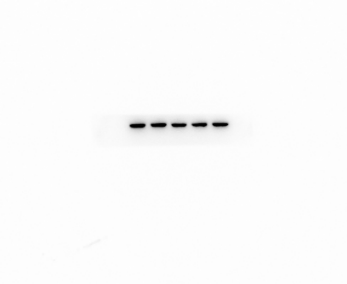

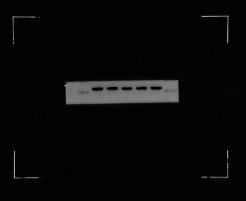


Actin


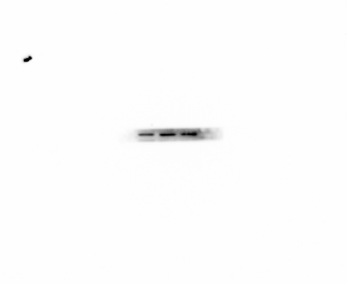

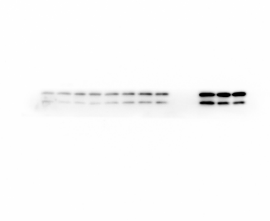
**Blots in Figure 6 A**


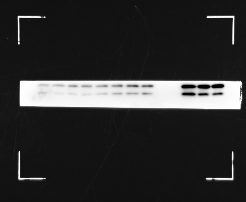

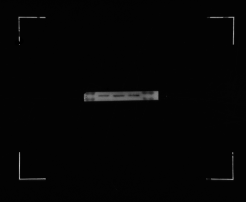
 P62 LC3


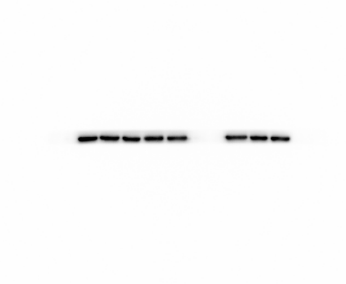

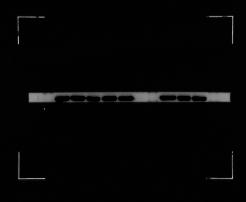

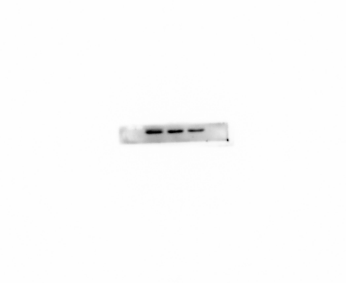

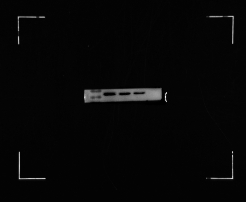


ATG5 GAPDH

**Blots in Figure 6 B**


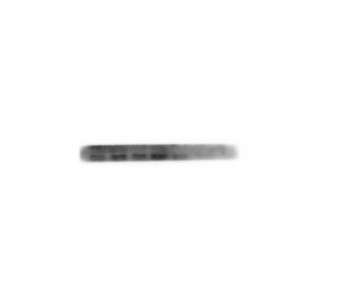

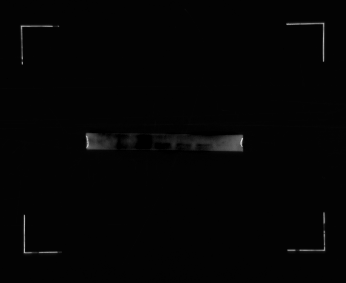

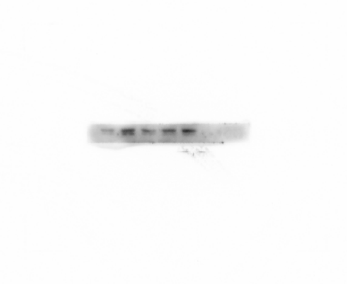

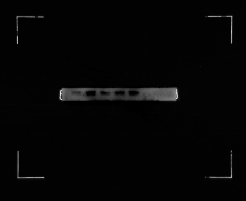
C-caspase9 Bcl-2


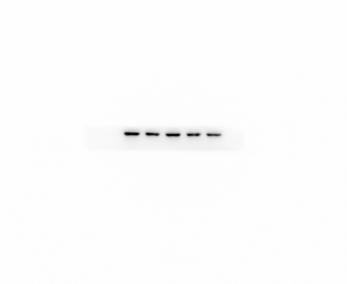

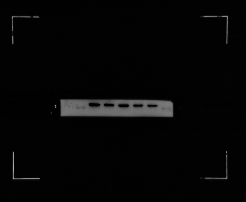

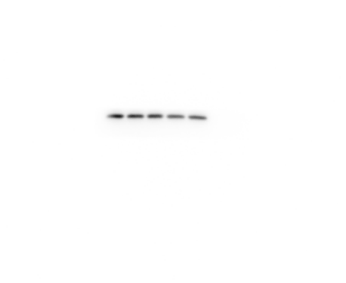

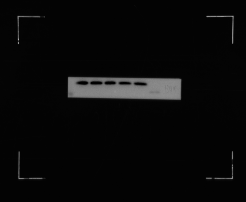


Bax actin
